# CGDV: a webtool for circular visualization of genomics and transcriptomics data

**DOI:** 10.1186/s12864-017-4169-5

**Published:** 2017-10-24

**Authors:** Vineet Jha, Gulzar Singh, Shiva Kumar, Amol Sonawane, Abhay Jere, Krishanpal Anamika

**Affiliations:** 1 Labs, Persistent Systems Limited, Pingala – Aryabhata, Erandwane, Pune, 411004 India; 2 Present address: Bioinformatics Center, Pune University, Ganeshkhind, Pune, Maharashtra 411007 India

**Keywords:** Circular diagram, Visualization, Web circos, Genomics and transcriptomics data visualization

## Abstract

**Background:**

Interpretation of large-scale data is very challenging and currently there is scarcity of web tools which support automated visualization of a variety of high throughput genomics and transcriptomics data and for a wide variety of model organisms along with user defined karyotypes. Circular plot provides holistic visualization of high throughput large scale data but it is very complex and challenging to generate as most of the available tools need informatics expertise to install and run them.

**Result:**

We have developed CGDV (Circos for Genomics and Transcriptomics Data Visualization), a webtool based on Circos, for seamless and automated visualization of a variety of large scale genomics and transcriptomics data. CGDV takes output of analyzed genomics or transcriptomics data of different formats, such as vcf, bed, xls, tab limited matrix text file, CNVnator raw output and Gene fusion raw output, to plot circular view of the sample data. CGDV take cares of generating intermediate files required for circos. CGDV is freely available at https://cgdv-upload.persistent.co.in/cgdv/.

**Conclusion:**

The circular plot for each data type is tailored to gain best biological insights into the data. The inter-relationship between data points, homologous sequences, genes involved in fusion events, differential expression pattern, sequencing depth, types and size of variations and enrichment of DNA binding proteins can be seen using CGDV. CGDV thus helps biologists and bioinformaticians to visualize a variety of genomics and transcriptomics data seamlessly.

## Background

Advancement in Next Generation Sequencing (NGS) technology has led to generation of unprecedented amount of data of different forms. Interpretation of large scale NGS data is complex and challenging. Visualization is one of the means to interpret NGS data and it plays crucial role in data analysis. Circular diagrams are very useful to view large scale data and their inter-relationship on a single frame. There are various web based tools available to visualize data in a circular view (Table 1). Online Circos (http://mkweb.bcgsc.ca/tableviewer/) which is based on Circos [1] is a webtool for visualizing data in a circular view but it requires detailed knowledge on how to use Circos. Tools such as CiVi [2] can only handle specified genomics data and is limited to plotting data from microbial genome. Another webtool, CliCo FS [3] only supports gene bank file. For other types of file it is not automated and hence user needs to format the file before the upload. Moreover, ClicO FS is visualization driven rather than data type driven. Additionally, multiple clicks are required before generating the plot. There are other desktop based applications such as J-Circos [4] which needs to be installed before running it. Moreover, J-Circos does not support all types of genomics and transcriptomics data formats and supports limited set of model organisms. Hence none of these tools support automated, guided, and a variety of genomics and transcriptomics raw output file to conveniently interpret data in a form of circular visualization, particularly for biologists with no or minimal knowledge on Circos installation and usage.

**Table 1:** Comparison of CGDV with other available web tools

Features	CIVI	ClicO^FS^	Circos Table Viewer	CGDV
Automated	✓	x	✓	✓
Manage raw output of various NGS data analysis tools	x	x	x	✓
Easy access to data and results	✓	✓	✓	✓
Prepackaged karyotype for multiple model organisms	x	x	✓	✓
Seamless upload and visualization of various genomics and transcriptomics data	x	x	x	✓
Generic data format support	x	✓	✓	✓

For generating any plot using Circos [1], karyotype file is must which defines basic information such as length of the chromosome/s of the reference genome or contigs’ length, its size, colour and appropriate labeling for each chromosome or contig. Another file required is configuration (config) file which contains information on how to visualize the data based upon its content. It is complex to make a config file as user needs to thoroughly understand the data, content and different possible visualization options.

**Fig. 1:**
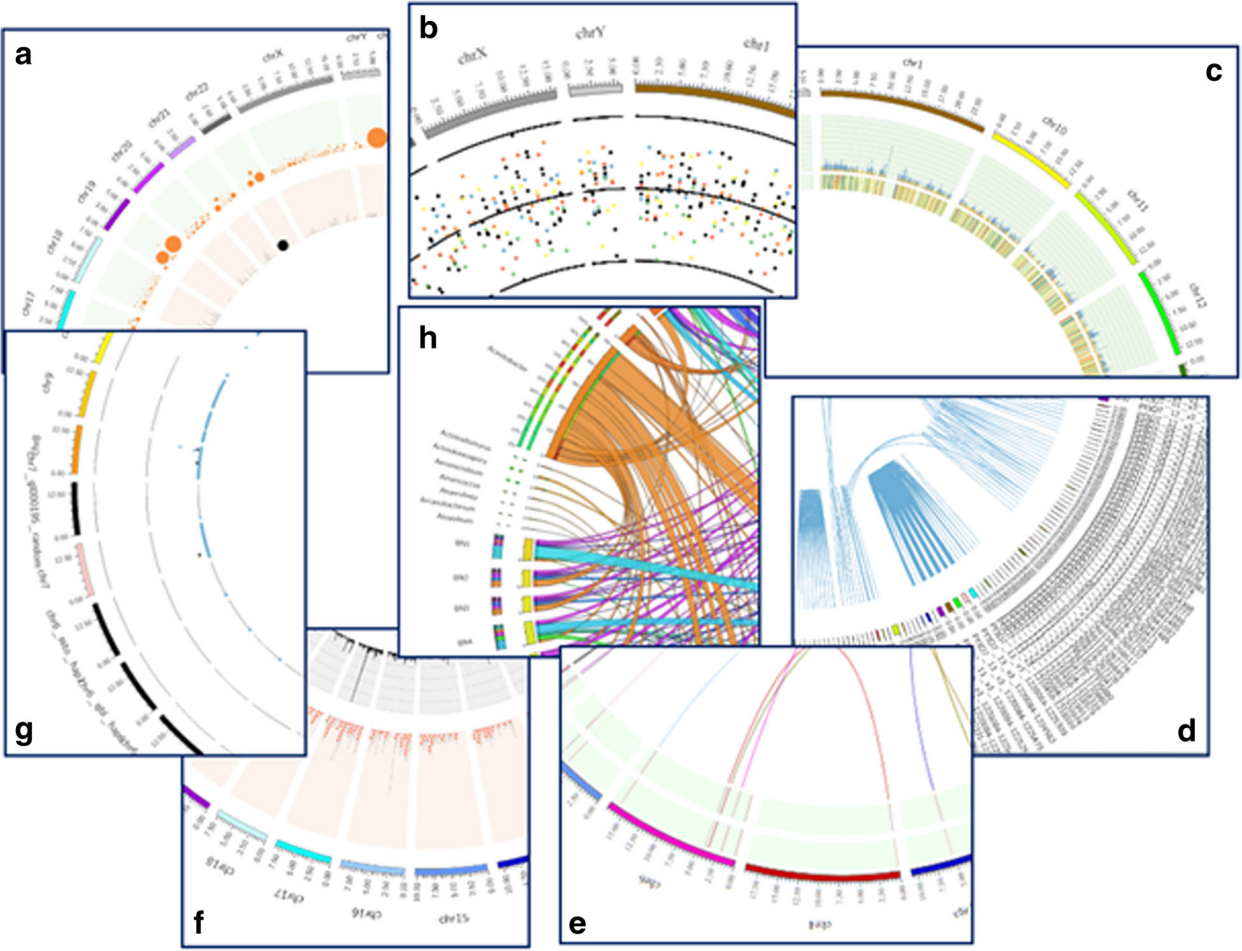
Various circular figures generated by CGDV for genomics and transcriptomics data. **a** This figure represent amplification (orange dots) and deletion (black dots) from raw output of CNVnator tool. The size of the circles represents relative size of the duplications and deletions at each location. **b** This figure represents data from a BED file. Each point represents the value per coordinate from a given sample. Black line represents mean value of the data. **c** This figure represents output of analyzed ChIPSeq data. Heatmap in the inner track represents fold-enrichment value of the peaks. The outer track is a histogram displaying tags with *p*-value. **d** This figure represents homologous region in genome from BLAST output in tabular format. **e** This figure represents gene fusion event result which is the output of STAR-Fusion and/or FusionInspector. The tracks are heatmaps representing Jffpm value (outer track) and Sffpm value (inner track). The links are the position of gene fusion events between chromosomes. **f** This figure represents data of a Variant Call Format (VCF) file which is output of tools such as GATK (https://software.broadinstitute.org/gatk/) and SAMTools (http://samtools.sourceforge.net/). Innermost track represents depth of variations and middle track represents SNPs and INDELs in black and red dots respectively. **g** This figure represents gene/isoform expression FPKM values from Cuffdiff output. Each gene/isoform FPKM values is plotted against various condition as dots. **h** This figure represents numerical data from a tab limited matrix

We have developed CGDV a wrapper around Circos which provides automated and guided generation of circular visualization of large scale genomics and transcriptomics data in a very seamless way. CGDV not only provides prepackaged karyotype files for various model organisms but also generates config file based upon the genomics and transcriptomics data provided by the user. CGDV takes standard raw output file of most of the genomics and transcriptomics data as input to generate data specific circular visuals in SVG and PNG formats (Fig. 1).

## Implementation

CGDV runs on apache web server. Web interface of CGDV requires input file along with other parameters such as user E-mail id (optional, can be run as a guest user), model organism, data type for which user would wish to create circular diagram. It extracts relevant information from input file and creates configuration and data files. Karyotype information of standard genome is stored in a SQLite database. As per selection of the model organism, specific karyotype details are fetched from the database. Using configuration files, data files and karyotype file, CGDV runs Circos [1] in the background and creates circular diagram for a given input file. CGDV generates images in SVG and PNG formats. If user provides E-mail id, output is archived for 15 days from the date of submission and deleted afterwards.

## Results and discussion

CGDV supports variety of genomics and transcriptomics data types (Table 2):

- CNVnator output: CNV (copy number variation) detected from CNVnator tool [5] can be uploaded for viewing insertion and deletion events. Deletions and amplifications are represented in black and orange circles respectively. Size of the circle is relative to the size of CNVs. This plot shows bird’s eye view of large structural variation present in the genome which is otherwise difficult to visualize in the genome viewer (Fig. 1a). User can filter the data based upon *p*-value before generating the plot.
- BED (Browser Extensible Data): Any type of data in BED format can be plotted in CGDV. For example, expression data in a BED format enables user to view and analyze expression of multiple genes of a genome. A maximum of 12 columns of the BED file can be plotted in the form of different colored dots. The middle black line represents the mean value. This image helps user to see the relative pattern on each location of the genome across samples (Fig. 1b).
- ChIPSeq output: Peaks detected from MACS [6] tool can be viewed in CGDV. Tag density of peak at each location is represented by histogram with its *p*-value (colour range with lower to higher *p*-values are represented in this order: violet, blue, green, yellow, orange, red). The fold enrichment of each peak is represented with heatmap. This circular plot helps user visualize genome-wide enrichment profile of DNA binding protein(s) of their interest (Fig. 1c). User can filter the data based upon number of tags, *p*-value and fold enrichment before generating the plot.
- BLAST output: BLAST [7] output file containing information on homologous regions/sequences can be plotted in CGDV. The homologous sequences/regions having 90% or more identity are linked to show conservation between them (Fig. 1 D). User can filter the data based upon e-value, identity, minimum hit length and score before generating the plot.
- Gene fusion output: Gene fusion event detected by FusionInspector (https://github.com/FusionInspector) can be easily viewed in CGDV. Inter and Intra gene fusion events are shown by links. FFPM value denotes fusion fragments per million total RNA-seq fragments. FFPM value is useful for filtering false positives. Those fusions showing up with FFPM values < ~0.2 are often false positive and hence we used FFPM value 0.5 and above. The color intensity in the outermost track shows Jffpm (Junction ffpm) while the inner track shows Sffpm (Spanning ffpm) values of reads. Higher color intensity bars in the two tracks suggest more number of reads supporting the fusion events. This image helps user visualize gene fusion events as links between genes with number of reads supporting fusion event (Fig. 1e).
- VCF (Variant Call Format): VCF file containing variations such as SNP (single nucleotide polymorphism) and InDel (insertion and deletion) with their respective sequencing depth can be visualized by CGDV. SNPs and InDels are represented in- Black and Red dots respectively and sequencing depth is represented as bar plot. Image generated from VCF file provides a holistic view of variation density in the genome, which sometimes is not captured in the genome browser (Fig. 1f). User can filter the data based upon read depth and quality of the data before generating the plot.
- Cuffdiff output: Gene expression detected by Cufflinks package [8] can also be plotted by CGDV. A maximum of 12 samples (in 12 different columns in a file) can be plotted with following colored dots: violet, indigo, blue, green, yellow, orange, red, brown, gold, gray in which violet dot represents the data in the first column and gray dot represents the data in the 12th column. This circular plot will help user in understanding differential expression of genes at global level in the sample data set (Fig. 1g). User can filter the data based upon *p*-value before generating the plot.
- Matrix link file: A matrix file containing data, a maximum of 150 tab separated columns can be plotted by CGDV. For example, different bacterial population in different conditions or locations can be plotted to display relationship between them. Image generated using matrix link file displays relation between the data in a different rows and columns by connecting them with links (Fig. 1h).

**Table 2:** CGDV supported data types, corresponding file formats and description of the plot

S.No.	Data type	File format	Circular plot
1	VCF	vcf version 4.1	SNP and InDel with their sequencing depth
2	CNVnator output	raw output of CNVnator	Amplification and deletion with their size
3	ChIPSeq	raw output from MACS in XLS format	Peaks and tag density with their *p*-value
4	Gene fusion output	raw output from fusion inspector	Links between various genes which are fused together with color intensity based upon number of reads supporting each fusion event
5	Cuffdiff output	raw output from Cuffdiff	FPKM values per gene /isoform
6	BED	Extended BED upto 12 data columns	Expression values per genome coordinate
7	Matrix-links	Data in a matrix format	Links between data in the row and column
8	BLAST output	BLAST output data in a tabular format (BLAST run with –m8 option)	Links between similarity among homologous sequences

## Conclusion

CGDV is an automated and easy to use web application for circular visualization of a variety of genomics and transcriptomics data. It supports output formats of most of the genomics tools, which, makes it a biologist friendly powerful tool for data visualization and interpretation. Our application not only supports micro-organism such as bacteria and fungi genome, but also supports large organisms such as human and mouse genome. Based upon user’s request more such karyotype files can be added to increase the diversity of model organisms.

## Availability and requirements

- Project name: CGDV
- Project home page https://cgdv-upload.persistent.co.in/cgdv/
- Operating system(s): Platform-independent
- Programming language: Python, Perl
- License: This web tool is free to all users without login requirement

## Abbreviations

BED: Browser extensible data
CGDV: Circos for genomics data visualization
CNV: Copy number variation
InDel: Insertion and deletion
NGS: Next generation sequencing
VCF: Variant call format
